# LeafFusionNet: a hybrid deep learning approach for robust plant disease detection

**DOI:** 10.3389/frai.2026.1773329

**Published:** 2026-03-25

**Authors:** Srijani Das, G. Prethija, C. Sudha

**Affiliations:** School of Computer Science and Engineering, Vellore Institute of Technology, Chennai, Tamil Nadu, India

**Keywords:** attention, Gabor filter, GradCAM, plant disease, PlantVillage dataset, transformer

## Abstract

**Introduction:**

Crop diseases have to be diagnosed early to save crop yields and food safety. Commonly used conventional practices are time-consuming and likely to involve human error. Automatic plant disease detection is a highly efficient technology that has been brought into existence by the development of deep learning and computer vision technology and can effectively detect the signs of diseases in plant species. Sustainable agriculture and early intervention depend on accurate and interpretable detection of plant diseases.

**Methods:**

This study introduces a hybrid model based on deep learning techniques that effectively identifies and categorizes leaf diseases. The proposed model, LeafFusionNet, incorporates Convolutional Neural Network (CNN) and Vision Transformer (ViT) with an efficient attention module, LeafTAM (Leaf Texture Attention Module), to effectively capture both global and local information. This architecture is enhanced by the addition of a Gabor filter layer before the CNN-ViT fusion, hence augmenting the model’s ability to extract physiologically relevant texture features.

**Results and discussion:**

The model was trained and validated on the Plant Village dataset. This model attained an accuracy of 99.33%, 99% precision, recall, and F1 score, demonstrating strong generalization on new data when compared with the state-of-the-art models. This proposed hybrid model can be utilized to develop a strong agricultural diagnostics system. These results point to the possibility of designing a powerful, interpretable system by utilizing transformer-based vision modules, Gabor filters, and explainability systems such as Grad-CAM.

## 1 Introduction

Roughly 40% of the total crops grown around the world are destroyed every year at the pre-harvest stage due to pest infestations (Global Burden of Crop Loss, 2019). According to the loss estimates made by the Punjab Agricultural University in India, plant diseases alone are responsible for 26% crop losses, leading to damages of over INR 290 billion annually (Shukla et al., 2022). About 5,000 of the 30,000 plant diseases documented worldwide have been reported in India. Accurate and interpretable plant disease detection is thus critical for sustainable agriculture and early intervention.

With the evolution of computer vision technology and with the emergence of deep learning, automated plant disease detection systems have been developed as one of the most effective solutions, which applies the image-based analysis to identify the symptoms of the disease in number of plant species. Many model architectures, preprocessing strategies, and benchmark datasets have been explored that maximize classification accuracy, model robustness, and computational efficiency.

Convolutional Neural Networks (CNNs) and Vision Transformers (ViTs) have gained importance due to their remarkable ability to learn contextual and spatial patterns in plant images. CNN-based methodologies have demonstrated exceptional performance on benchmark datasets such as PlantVillage, achieving classification accuracies of up to 96% (Sahana et al., 2025).

Ouamane et al. (2025) introduced a Vision Transformer model that has shown better results due to the model’s global dependencies and achieved better results than state-of-the-art performance. Chai et al. introduced the PlantAIM model, which integrates CNNs and ViTs, and achieves an accuracy of 99.66% on real-world datasets, demonstrating robust performance (Chai et al., 2025).

Ensuring high classification accuracy across a wide range of diseases, adapting models to new environmental settings, and enhancing interpretability to foster user trust among farmers are even challenging despite these innovations. These demands require models to combine learned and hand-engineered features and to provide interpretable outputs. The main contributions to this work are:

- A zero-parameter Gabor filter that uses depth wise convolution to extract orientation-dependent texture patterns is introduced. This provides explicit texture priors that are relevant for plant disease patterns such as lesions, mottling, and streaks.
- A dual-stream hybrid architecture combining CNN and vision transformer is introduced. CNN extracts local features, ViT identifies long-range interactions across the surface of a leaf.
- A dual attention model LeafTAM (Leaf Texture Attention Module) integrates channel-wise excitation and Gabor texture features with spatial attention to highlight patterns associated with the disease and removes background noise.
- LeafFusionNet is a feature fusion architecture that combines CNN features, LeafTAM features, and ViT embeddings into one representation, which captures fine features and broader structural patterns. It maintains high discriminative performance and achieves high accuracy.
- Furthermore, Grad-CAM (Gradient Weighted Class Activation Mapping) visualizations are employed to elucidate the interpretive rationale of the proposed paradigm.

The proposed architecture does this by combining Gabor filter features, CNN features, LeafTAM features, and ViT embedding’s into one reliable representation. It shows complex features, including underlying structural patterns.

## 2 Related works

Plant disease detection has undergone a remarkable systematic evolution with modern deep learning methods of hybrid architecture, attention, and explain ability methods like Grad-CAM and GRAD-CAM++. This section reviews the current state-of-the-art methods and identifies the gaps that motivate the proposed LeafFusionNet model.

### 2.1 CNN-based approaches for plant disease detection

Initial deep learning methods used mostly Convolutional Neural Networks (CNNs) in order to extract spatial hierarchies and disease-specific markings in plant images. Sahana et al. (2025) used CNN architecture and reported an accuracy of 96% on the Plant Village dataset, indicating its ability to capture spatial disease patterns. Chowdhury et al. employed CNNs for feature extraction and attained an accuracy of 85.31% over 14 disease classes across 17,430 images. This highlights the ability of CNNs in dealing 78 with large-scale regional datasets (Chowdhury et al., 2025).

Various ensemble methods were devised for improving performance over a wide range of datasets. A hybrid architecture theta that combines the features generated by combining the InceptionResNetV2, MobileNetV2 (Dong et al., 2020), and EfficientNetB3 (Sogarwal et al., 2025) models is developed. This hybrid has shown outstanding performance across the PlantVillage, PlantDoc, and FieldPlant datasets (Zubair et al., 2025). A hybrid model that amalgamates InceptionNet and XceptionNet was developed by Shafik et al. (2025). This model achieved almost perfect accuracy on six datasets, including PlantVillage dataset, thereby increasing feature extraction and reducing the risk of overfitting. Similarly, an Attentive Self-supervised Contrasting Learning (ASCL) framework (Yilma et al., 2025) incorporates both attention and self-supervised contrastive learning and a ResNet (Residual Network) backbone, reached 93.50% on Apple and 83% on Wild Tomato datasets, using contrastive learning and channel-wise attention as the training method.

The DynLeafNet framework was developed by Verma et al., (2025), replacing traditional static neural networks with a dynamic residual architecture for the classification of plant diseases. Instead of using fixed computation paths during inference, DynLeafNet uses dynamic structural and parametric adjustments during inference, allowing the network to adapt to the difficulty of each input image. By using this adaptive approach, we achieved state-of-the-art accuracy of 98.33% on the 38-class PlantVillage dataset with fewer FLOPs and parameter counts. The study also integrates Explainable AI (XAI) techniques, including Grad-CAM and LIME, to provide transparent visualizations of the textural regions driving the model’s decisions. This bridges the gap between high-performance deep learning and practical, interpretable agricultural diagnostics, and is therefore achieved.

An investigation by Gopalan et al. (2025) shows that ResNet152 was applied for detecting corn disease, with a test accuracy of 93.69% and improved model interpretability. Equally, Karim et al. heat mapped attention to a trained MobileNetV3 Large model running on an edge device (Nvidia Jetson Nano) to monitor grape leaf diseases in real-time, which can help decision-makers understand the reasoning behind the model’s behavior and performance using Grad-CAM (Karim et al., 2024). Albahli et al. proposed a novel and high-quality lightweight framework called AgriFusionNet that is used to diagnose plant diseases through the combined use of RGB images, multispectral drones, and the input of environmental sensors with IoT. Their model is built on an EfficientNetV2-B4 backbone, with Fused-MBConv blocks and Swish activation functions to improve gradient flow. The model was also scored at 94.3% accuracy in a large, multi-modal dataset, and it has a significantly lower number of parameters than the standard Vision Transformers and a very small inference latency of 28.5 ms (Albahli, 2025).

Although these CNN-based methods are recognized as effective models on benchmark datasets, they lack in capturing long-range dependence and global contextual information, which is significant to distinguish subtle disease patterns akin to those observed on entire leaf surfaces.

### 2.2 Vision transformer (ViT) based approaches

Vision Transformers (ViTs) have been recognized for plant disease detection because of their capability to simulate long-range relationships. Ouamane et al. trained a plant village dataset on Vision Transformer with a set of hyper parameters. This has shown better results than CNN models such as VGG19 (Visual Geometry Group) and AlexNet in accuracy and computational efficiency (Ouamane et al., 2025). Few-shot learning has also been another direction that has been important. PlantCaFo is a 3-step ViT-based model, and it demonstrated enhanced performance in data-scarce conditions and reached an ultimate accuracy of 94.23% on the PlantVillage dataset. It also adopted the Feature Attention modules to reduce the background noise and maximize the discriminative localization (Jiang et al., 2025).

Moreover, attention mechanisms have become vital to improving models’ performance by focusing on disease-relevant regions and suppressing background noise. Qian et al. incorporated self-attention mechanism into a transformer model for detecting maize disease. Their model has achieved 98.7% accuracy with lower computational cost than CNNs (Qian et al., 2022). The model outperformed CNN baselines, including VGG11 (97.9%), EfficientNet-B3 (91.6%), and ResNet50 (96.6%).

Isinkaye et al. trained Variational Autoencoders (VAEs) on high-dimensional data in combination with ViTs and obtained 93.2% accuracy with a variety of species. This hybrid model was able to achieve both global and local dependencies and is indicative of multi-module architectures being versatile (Isinkaye et al., 2025).

While these attention mechanisms enhance feature refinement, they are typically applied to learned CNN or transformer features rather than to explicitly texture-enhanced representations. None of the existing approaches integrate dual attention (channel and spatial) specifically with hand-crafted texture features to create a texture-aware attention pathway.

### 2.3 Hybrid CNN-ViT architectures

Research has focused on hybrid architectures in recognition that CNNs (local feature extraction) and ViTs (global contextual modeling) offer complementary strengths. A new ensemble framework combining VGG16, Inception-V3, and DenseNet201 with ViT was proposed by Aboelenin et al. (2025) as an approach for achieving 99.24% accuracy with apple datasets and 98% accuracy with corn datasets. In comparison with single-architecture models, the hybrid approach provided better classification performance when CNN-extracted local features were integrated with ViT-captured global dependencies.

Chai et al. (2025) introduced the PlantAIM model, which integrates CNNs and ViTs, and performs better than single-model architectures with an accuracy of 99.66% and, demonstrating robust performance on real-world datasets. The Global Local Feature Aggregation (GLFA) layer refines CNN and ViT features extracted independently by addressing global patterns and localized manifestations. Based on Grad-CAM visualizations, PlantAIM is capable of capturing important disease symptoms and leaf patterns, which provides practical interpretability for agriculture. PlantAIM incorporated Grad-CAM to visualize both crop-specific patterns and disease-specific symptoms, demonstrating that the hybrid model focuses on biologically relevant features. The interpretability provided by Grad-CAM proved essential for validating model behavior and fostering adoption among farmers and agronomists.

A hybrid architecture called ConvTransNet-S to bridge the gap between laboratory accuracy and real-life agricultural performance is presented by Jia et al. (2025). It incorporates a Lightweight Multi-Head Self-Attention (LMHSA) and a Local Perception Unit (LPU) to preserve translation invariance while maintaining microscopic lesion detail and leaf context. The 98.85 percent accuracy on the PlantVillage benchmark was reported by ConvTransNet-S when tested in a complex field environment. The Eff-Swin-HGSO (Higher Gravitational Search Optimization) framework is a multi-stage hybrid approach that uses EfficientNetV2B0 as a feature extractor to extract local features and uses Swin Transformers to extract the global context. It utilizes a metaheuristic optimization algorithm based on Henry’s gas solubility to reduce the high-dimensional feature space (Alsakar et al., 2026). It applies an attention-based fusion mechanism and uses an RBF-SVM (Radial Basis Function Support Vector Machine) classifier to achieve an accuracy of 99.2 percent. The experiments are carried out in the PlantVillage dataset.

There are still several hybrid architectures that employ two-stream fusion (CNN + ViT) and do not explicitly incorporate hand-engineered texture priors that could enhance the model’s sensitivity to fine-grained diseases like lesions, venation abnormalities, and mottling, despite these advances.

### 2.4 Gabor filters and texture-based feature extraction

Gabor filter and texture-based feature extraction have been considered with the aim of highlighting low-level features such as venation and lesion boundaries. Nashrullah et al. (2021) applied an SVM classifier to Gabor and color filters to identify tomato leaf diseases with a classification accuracy of 90.37%. Singh and Kumar (2024) proposed a high-performing diagnosing model to detect plant leaf disease with the help of Gabor filters and a Constitutive Artificial Neural Network (CANN) architecture. It begins by pre-processing the image using Gabor filters to identify the features of the textures, which are needed in the distinction between normal and sick plant leaves. Followed by that, the Adaptive Convex Clustering (ACC) algorithm was used as an image segmentation method to isolate regions of interest. In the extraction of features, the model uses Fast Fourier Transform (FFT) and Continuous Wavelet Transformation (CWT) to extract both frequency and spatial information of the segmented images. This model demonstrated a high accuracy of 99.89% for the pumpkin leaf disease dataset. By integration of Gabor filters, the model increased sensitivity to fine-grained texture changes and avoided over fitting.

Kwabena et al. (2020) introduced a Gabor Capsule Network (Gabor CapsNet) with an accuracy of 98.13% on tomato and 93.33% on citrus, which achieved higher results than CNN baselines, as well as a Capsule Network model. This approach proved to be resistant to deformed and invisible images, which makes it suitable to be applied in the real world, but the complexity of implementing it and the significant volume of data required to train the Gabor CapsNet model were observed.

Explain ability has become increasingly important for building trust in AI-based agricultural systems. Gradient-weighted Class Activation Mapping (Grad-CAM) has emerged as the primary technique for visualizing which regions of input images influence model predictions. While Grad-CAM has been widely adopted for interpretability in plant disease detection, existing applications visualize attention on standard learned features. No research has been done by combining Grad-CAM with texture-enhanced attention modules to specifically validate whether models are attending to texture-based disease indicators such as lesion boundaries, venation patterns, and mottling structures. A combination of these works shows how plant disease detection emerged from traditional CNNs and human-designed feature extractors to advanced hybrid transformers and attention infrastructures (Table 1).

**Table 1:** Summary of existing methods for plant disease detection.

S. No	Author/reference	Techniques used	Accuracy, dataset used	Advantages	Limitations
1	Sahana et al., 2025	CNN	96% PlantVillage dataset	Achieves accuracy with strong generalization on diverse plant species	No explicit handling of complex backgrounds or real-world conditions
2	Chowdhury et al., 2025	CNN	85.31% Plant village dataset	Cost-effective model for local farmers, automates feature extraction	Limited to 3 crops, lacks local dataset diversity
3	Zubair et al., 2025	Inception ResNetV2, MobileNetV2, and EfficientNetB3.	60% on PlantDoc 83% on FieldPlant	Better performance in diverse datasets through ensemble learning	High complexity may limit real-time use
4	Verma et al., 2025	DynLeafNet	98.33 on plant village dataset	Adaptive Computational Efficiency	Decision-Gate Overhead, Inference Latency Instability
5	Ouamane et al., 2025	Vision Transformer (ViT) model	98.75% on BananaLSD Dataset	Model compactness, Generalization	Training complexity, Resource-intensive
6	Jiang et al., 2025	PlantCaFo, Vision Transformers (ViT)	93.53% on PlantVillage Dataset and 72% on Cassava Dataset	Effective few-shot learning model using ViT for data-scarce scenarios	Latency, Computational Efficiency
7	Aboelenin et al., 2025	VGG16, InceptionV3, DenseNet201 + ViT	99.24% for the apple dataset 98% for the corn dataset	Combines global and local features for high classification accuracy	Need large datasets and computational resources.
8	Jia et al., 2025	ConvTransNet-S	98.85% on PlantVillage dataset	Robustness to Background Noise	Accuracy Drop in Complex Scenes, Structural Complexity
9	Isinkaye et al., 2025	Variational Autoencoders (VAEs) and ViTs	93.2% Images extracted from new Plant diseases dataset on kaggle	Combines global and fine-grained feature learning effectively	Minor misclassifications in diseases due to inter-class similarity
10	Alsakar et al., 2026	Eff-swin-HDSO	99.2% on PlantVillage dataset	Effective Dimensionality Reduction	Optimization Overhead, Lack of Direct Spatial Interpretability
11	Qian et al., 2022	Transformer architecture with self-attention mechanisms	98.7% PlantVillage + taken by mobile phones in the natural environment	Outperforms CNNs with high accuracy and low FLOPs	Increased complexity
12	Yilma et al., 2025	ASCL, self-supervised contrastive learning, with ResNet	93.50% on Apple dataset 83.00% on Wild Tomato	Self-supervision, Generalization, No need for annotation	Domain-sensitive, Dataset-specific performance
13	Nashrullah et al., 2021	Gabor Filters and color filters, SVM, RBF	PlantVillage Dataset. High sensitivity (90.37%), early detection, F1: 90.34%. (Specificity, AUC: 94.60%)	Better accuracy	Technically demanding and dependent on input image quality; requires domain expertise
14	Singh and Kumar, 2024	CANN + Gabor Filters	99.89% on Pumpkin Dataset	Highest accuracy with strong generalization.	Complex architecture is not suited for low-resource settings
15	Kwabena et al., 2020	Gabor Capsule Network (Gabor CapsNet)	98.13% for tomato and 93.33% for citrus datasets	Robust even on unseen/deformed data, fast convergence	Complex to implement, requires large datasets
16	Gopalan et al., 2025	ResNet152 + Grad-CAM	93.69% cumulative dataset was imported from the Kaggle repository	High test accuracy (93.69%) with explainability for end users.	Heavy models, sensitive to lighting and background variation.
17	Karim et al., 2024	CNN + Grad-CAM (Edge device)	99.42% Grapevine Disease Dataset	Real-time monitoring on Nvidia Jetson Nano	Hardware limitation
18	Alhwaiti et al., 2025	YOLOv3, YOLOv4	97% with YOLOv3 model, 98% with YOLOv4 model on PlantVillage dataset	Real-time detection with high accuracy	Training is resource-intensive and struggles with data imbalance

### 2.5 Research gap

The literature review reveals several key trends, including hybrid CNN-ViT architectures that outperform single-model approaches by combining local and global feature extraction, attention mechanisms (Feature Attention, GLFA, channel-wise attention), improving the ability to interpret and refine features, and Gabor filters, which capture texture-based disease patterns, are primarily applied to traditional machine learning architectures rather than modern deep learning architectures.

Despite these advances, existing hybrid models do not explicitly integrate hand-engineered textures such as those captured by Gabor filters into attention-based deep learning pipelines. Incorporating Gabor filters addresses a fundamental theoretical gap by explicitly encoding texture priors that complement learned features. This is particularly important for diseases characterized by fine-grained texture patterns such as lesions, mottling, venation abnormalities, and surface streaks that may be underrepresented in purely data-driven feature learning.

This work combines these identified gaps into a unified framework that integrates Gabor filters as a zero-parameter preprocessing layer within a modern deep learning pipeline, which explicitly encodes orientation-dependent texture patterns. In addition, it applies dual attention mechanisms (channel and spatial) specifically to Gabor-enhanced features to create a texture-aware attention pathway and performs three-way feature fusion combining independent streams of CNN features, texture-attention-enhanced features (LeafTAM), and ViT global embedding’s. It also provides texture-aware interpretability through Grad-CAM visualization of the attention module to validate focus on texture-based disease indicators.

The proposed LeafFusionNet model incorporates these practices and combines global feature extractors (ViT), local feature extractors (CNN), and texture-aware attention (LeafTAM applied to Gabor-enhanced features). This architecture bridges hand-crafted and learned representations and maintains interpretability through Grad-CAM visualization, essential for deployment in precision agriculture.

## 3 Proposed method

**Figure 1:**
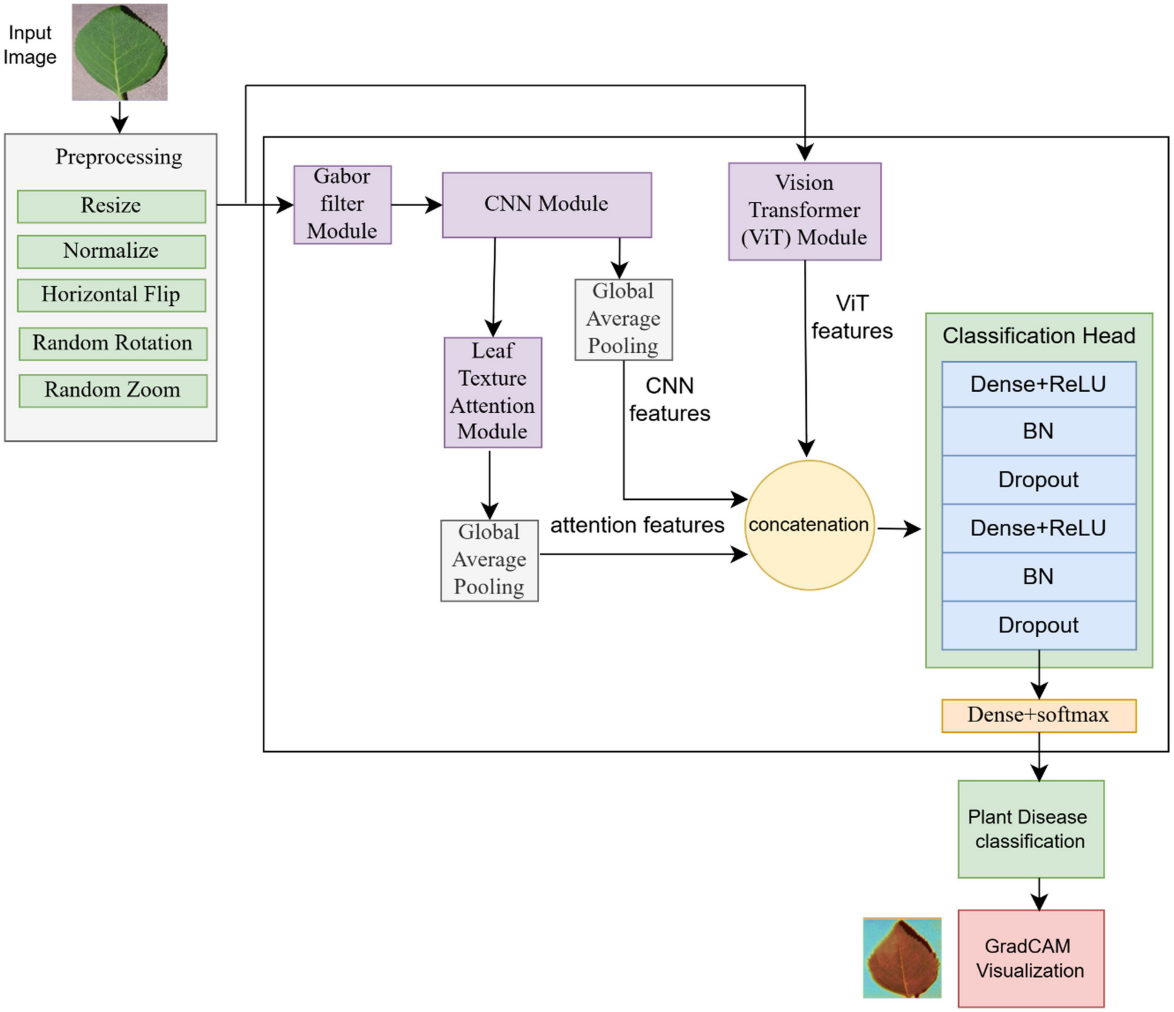
Methodology overview.

LeafFusionNet is a hybrid model that employs ViT, CNN, and LeafTAM architecture and is completed with the help of a handcrafted feature-extraction layer, i.e., Gabor Filter Layer, to focus on the leaf texture, such as venation, edges, and disease-specific surface patterns. The general approach of the planned model is presented in Figure 1. The pipeline consists of the processes of preprocessing of the input leaf images (resize, normalization, and data augmentation). The processed image has two parallel streams: (1) The original input to a pre-trained ViT Vision Transformer (ViT) to extract global features, (2) The original input into a Gabor filter module and the subsequent CNN-based local features extraction. The CNN module processes the Gabor-filtered features, and then they are refined by the Leaf Texture Attention Module (LeafTAM), which uses both channel and spatial attention processing. Three distinct feature streams are identified, namely CNN features (through Global Average Pooling), texture-attention features (LeafTAM output through Global Average Pooling), and ViT features, that make a single multi-scale representation. This composite feature representation is fed into a two-layer classification head using Dense +ReLU, Batch Normalization (BN), and dropout regularization, terminated by a Dense + Softmax final layer to classify the disease into 38 categories. The LeafTAM module is used with Grad-CAM visualization, which offers the option of visualizing the module with interpretative regions perplexed with the disease of interest on the input images.

### 3.1 Dataset used

The PlantVillage dataset consists of about 54,305 leaf images of both unhealthy and healthy categories of a wide variety of crops. The dataset comprises 38 class-specific directories, and each of the directories depicts a different combination of plant diseases (Mohanty et al., 2016). These colored images maintain important texture and color details needed in effective disease diagnosis. It has high-quality annotations and provides a variety of classes, which are suitable for creating powerful deep learning models in detecting plant diseases. The dataset has 38 classes, each of which corresponds to a single combination of plant species and disease status. It covers crops such as tomato, potato, maize, apple, grape, cherry, soybean, and peach with a wide range of disease types for plant disease classification. The dataset includes various disease manifestations such as fungal diseases (powdery mildew, rust, leaf spot, black rot), bacterial diseases (bacterial spot, bacterial blight), viral diseases (Mosaic virus, leaf curl virus) and pest damage (spider mites, leaf miners).

Despite the fact that the similarity of names of certain diseases may exist among plant species, the characteristic appearance usually differs based on the morphology of the host leaf, the pattern of the venation, and the distribution of colors. In this work, each class is defined with a combination of both plant species and type of disease. Thus, the model will automatically acquire plant-specific traits and disease features, which will decrease the chances of confusion between species.

**Figure 2:**
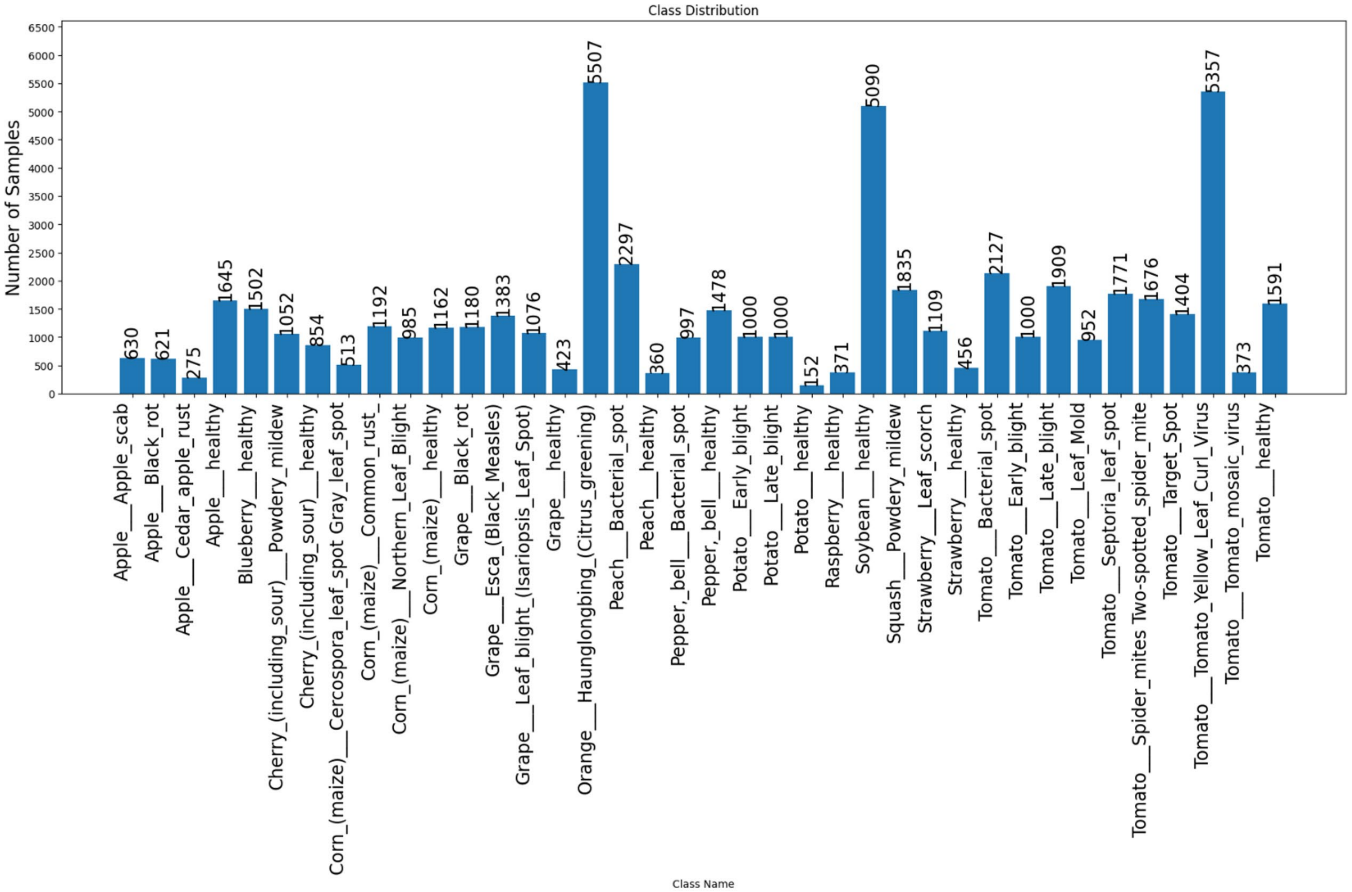
Class-wise distribution of images in the PlantVillage dataset.

The PlantVillage dataset employed in this study shows considerable class imbalance, which reflects real-world agricultural scenarios where certain diseases are more common than others and where few crops are extensively photographed. Figure 2 present Class-wise distribution of images in the PlantVillage dataset, showing the number of samples available for each crop–disease category. This imbalance is characteristic of real-world plant disease datasets where certain diseases are more frequently documented. The model treats each unique combination of (plant species + disease type) as a completely separate and distinct class, acknowledging that disease manifestations vary among host species.

**Figure 3:**
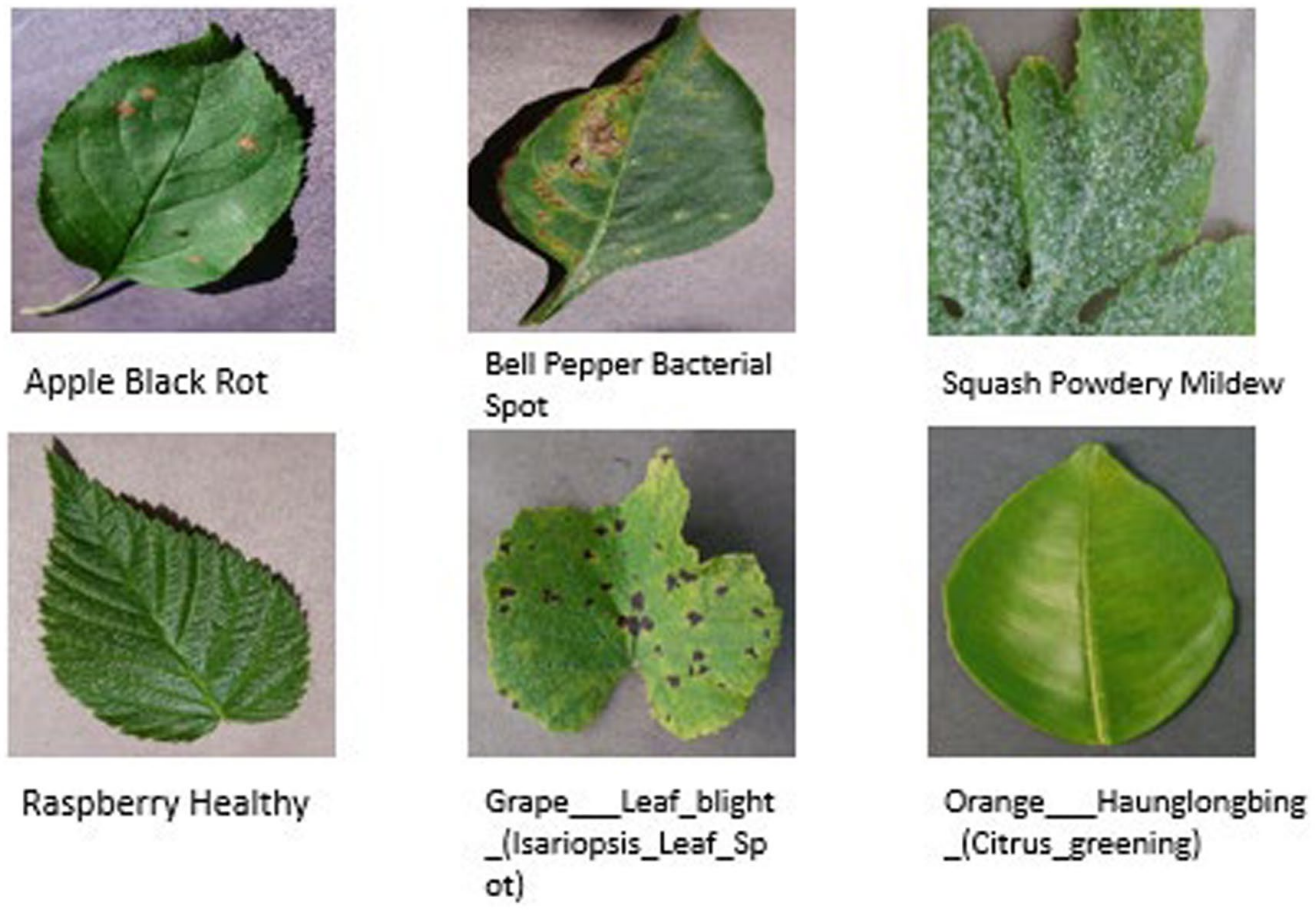
Sample images from PlantVillage dataset.

The data was split into training, validation, and test data to enable a thorough review of the model. To begin with, 20 percent of all the images were reserved as the test set through stratified sampling so that the proportions of classes remain the same. The rest of 80% of the data was split into training and validation sets, and 20 percent of the data was used in validation, which also uses stratified sampling. Figure 3 illustrates sample images from Plant Village dataset.

### 3.2 Preprocessing phase

These preprocessing steps include the resizing process, normalization, and augmentation, like horizontal flip, random rotation, and random zoom.

#### 3.2.1 Image resizing

All images were resized to 224 × 224 pixels so that the images could fit in the models as required. This standardization serves multiple purposes. It is compatible with the expected input dimensions of pre-trained ViT models, which makes it possible to use transfer learning from ImageNet effectively. In addition, it provides computational consistency across the entire pipeline. Furthermore, it has enough resolution to keep the details of critical disease symptoms while ensuring overall processing efficiency is maintained.

#### 3.2.2 Normalization

During training, the pixel values were normalized to the range [0, 1] from [0, 255] to improve the convergence of the model.

#### 3.2.3 Data augmentation

To avoid over fitting and for better generalization, data augmentation techniques such as random horizontal flipping, random rotation (upto 10%), and random zoom (upto 10%) are adopted.

Augmentation techniques overcome the drawback of limited samples in a few crops. This introduces variability in the training data, thereby simulating the variability that occurs in the real world with respect to leaf orientation, camera angles, and scale. Horizontal flipping simulates different leaf orientations

- Random rotation (±10%) to account for natural variation in how leaves are photographed, without unrealistic distortions to the images
- Random zoom (± 10%) to simulate variation in camera distance and leaf size

Importantly, these augmentations are applied before the Gabor filtering and ViT processing, which ensures that all feature extraction pathways benefit from the increased training data diversity.

### 3.3 Model architecture

LeafFusionNet can be explained as a hybrid model that integrates CNNs, ViTs, and a custom attention module named LeafTAM. It thereby takes advantage of both local and global feature representations and enhances interpretability and model attention. The main framework takes into consideration both local and global handcrafted features, with selective attention through various attention mechanisms.

#### 3.3.1 Gabor filter layer for texture enhancement

Plant diseases frequently manifest as orientation-dependent texture patterns, including venation abnormalities, lesion boundaries, streaks, and mottling. The incorporation of Gabor filtering is motivated by the fact that the symptoms of plant diseases are highly textual and orientation specific (Nabwire et al., 2022). Most of the infections appear in the form of spots, lesions, blights, or vein distortions that are different from normal tissue, mainly in their frequency and directional patterns at the location. Gabor filters are famous due to their joint spatial-frequency localization property, as well as due to their ability to detect edges, texture and orientation.

Different disease symptoms are demonstrated at different spatial frequencies. Early-stage bacterial spots and powdery mildew require high-frequency filters. To detect medium textures like developing lesions, spot patterns, mid-frequency filters are utilized. However, large necrotic lesions and late blight symptoms require low-frequency filters. A Gabor filter uses a filter bank with multiple frequencies, which allows detection of disease symptoms across different scales simultaneously. The theta (*θ*) parameter rotates the filter to detect edges and patterns at specific angles (0° detects vertical patterns, 45° detects diagonal patterns, and 90° detects horizontal patterns). Disease patterns often exhibit directional characteristics (Javidan et al., 2024). A circular spot with radial patterns, which can be detected in multiple orientations. Linear lesions following leaf veins can be detected with specific orientation filters. The combination of orientations is used to detect irregular multi-directional patterns. A unique texture is created by combining frequency and orientation responses. Healthy and diseased leaves have dramatically different texture characteristics. Usually, healthy leaves have a uniform, smooth texture with consistent vein patterns, and diseased leaves have spotted patterns (bacterial/fungal infections), irregular boundaries (lesion edges), color variations with texture changes, and vein discoloration with altered texture.

The model uses a special Gabor Layer on the input image with eight Gabor filters of dissimilar orientations, whose outputs are subsequently fed into the input feature map. Gabor filters are useful for extracting orientation and texture information, and in detecting patterns of fungal infection or mottled regions on leaves. Each Gabor kernel gabor (x, y) is computed as in Equation 1.


$$\text{gabor}(x,y)=\exp(-\frac{1}{2}\;\frac{x_{\theta }^{2}+\gamma^{2}y_{\theta }^{2}}{\sigma^{2}}\;)X\;\cos(2\pi \frac{x_{\theta }}{\lambda }+\psi )$$ (1)

Where, $x_{\theta }=x\cdot (\cos\theta )+y\cdot (\sin\theta ),$ $y_{\theta }=-x\cdot (\sin\theta )+y\cdot (\cos\theta ),$ θ denotes orientation, wavelength is represented by ʎ, *γ* represents aspect ratio, *σ* symbolizes the standard deviation of the Gaussian envelope, which provides spatial localization and prevents response to distant features and ψ denotes the phase offset that control filter’s frequency and spatial characteristics.

The multi-channel texture-enhanced image produced by this layer is passed to the CNN module.

#### 3.3.2 CNN module for local feature extraction

**Figure 4:**
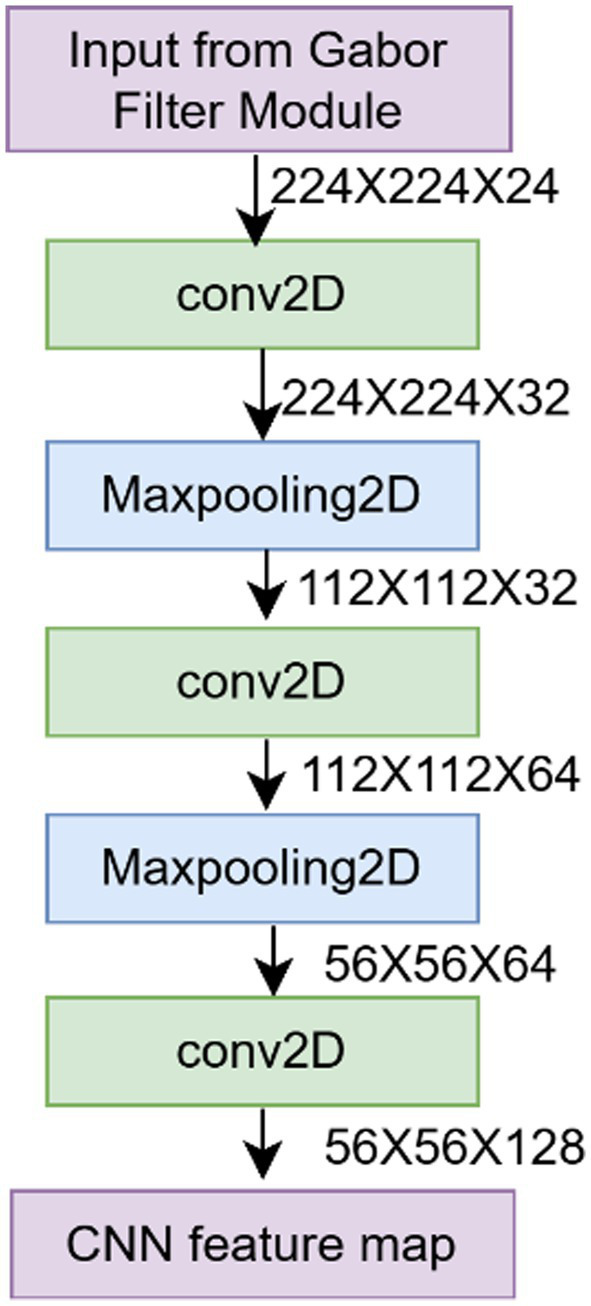
CNN module for feature extraction.

The CNN branch serves as the basis to capture localized spatial patterns such as edges, colour variations and leaf venation, which are essential for the detection of disease symptoms (Figure 4). This module extracts the features from texture-enhanced inputs, which are used as a robust representation.

CNN block consists of three convolutional layers with filter depths of 32, 64, and 128, respectively, as shown in Equations 2–4, followed by maxpooling layer for reducing the spatial resolution through retaining salient features. The increase in the depth of the filters enables the network to learn more and more complex and abstract features: the first layers (32 filters) learn simple features like edges and color changes, the next layers (64 filters) turn these into other more complex features like the venation in the leaves, the next levels (128 filters) learn high-level disease-specific patterns. This feature learning of hierarchical nature has been proven in various investigations to be effective in plant disease detection. Smaller kernel sizes (3 × 3) enable the network to acquire localized spatial structure without compromising parameter efficiency and stacking more convolutions of 3 × 3 size in an effective way for increasing the receptive field in subsequent layers.

ReLU (Rectified Linear Unit) activation functions are used because they are computationally efficient and can help avoid the vanishing gradient issues in back propagation (Banerjee et al., 2019). ReLU has proven to be the default activation function in CNN-based plant disease detection models because it provides non-linearity and provides sparse activation patterns, which enable the network to focus on the most informative regions of lesions and avoid activation of the background and thus model generalization. Max pooling layers are applied after each convolutional block to progressively reduce spatial dimensions while retaining the most salient features. This dimensionality reduction decreases computational requirements in deeper layers, provides translational invariance to small shifts in disease symptom position, and prevents overfitting by reducing the total number of parameters. ReLU encourages sparse activation, and it allows the network to concentrate on the most informative lesion regions and prevent activation of the background. The behavior is beneficial in the detection of plant diseases, whereby the pathological patterns do not usually cover a substantial space.

Let X *ϵ* R^HxWx3^ be the input image. The CNN branch sequentially computes the output feature as shown in Equations 2−4.


$$F_{1}=\text{MaxPool}(\text{ReLU}(\mathrm{Con}v_{3X3}^{32}(X)))$$ (2)

$$F_{2}=\text{MaxPool}(\text{ReLU}(\mathrm{Con}v_{3X3}^{64}(F_{1})))$$ (3)

$$F_{3}=\text{MaxPool}(\text{ReLU}(\mathrm{Con}v_{3X3}^{128}(F_{2})))$$ (4)

The output feature map is later used in both attention processing and global average pooling to produce compact local descriptors. The final feature map is processed using Global Average Pooling rather than flattening or global max pooling. GAP offers distinct advantages: it significantly reduces the number of parameters compared to fully-connected layers (mitigating over fitting risk), enforces correspondence between feature maps and categories (enhancing interpretability), and has been shown to improve generalization in image classification tasks. Global Average Pooling (GAP) is applied to the final feature map as shown in Equation 5.


$$\mathrm{CN}N_{\text{features}}=\mathrm{GAP}(F_{3})$$ (5)

#### 3.3.3 ViT module for global feature extraction

The localized receptive fields of CNNs have inherent limitations in modeling long-range dependency across entire leaf surfaces. Transformers overcome this shortcoming using self-attention mechanisms that calculate relationships between all spatial regions simultaneously, helping the model to understand more global patterns, such as the distribution of disease over large areas of a leaf. The use of a pre-trained ViT makes use of transfer learning from large-scale datasets (ImageNet) and provides powerful initial feature representations that decrease training time and improve generalization, which is even more important considering the limited size of plant disease datasets compared to the general image classification benchmarks (Hu and Guan, 2024).

To capture global contextual information, a pre-trained Vision Transformer is used. The input image is resized to 224 × 224 to match the transformer model’s expected input format, as given in Equation 6.


$$\mathrm{Vi}T_{\text{features}}=\mathrm{Vi}T_{\text{pretrained}}(\text{Resize}(X,224X224))$$ (6)

The ViT uses the input image by first dividing the image into a series of flattened patches that are linearly embedded and fed through a series of transformer layers. The model can capture long-range dependencies and spatial relationships using self attention mechanism as shown in Equation 7.


$$\text{Attention}(Q,K,V)=\text{softmax}(\frac{QK^{T}}{\sqrt{d_{k}}})\;V$$ (7)

Where:

- QK^T^ represents the similarity measure between query Q and Key K.
-
$\sqrt{d_{k}}$ represents a key vector dimension that stabilizes training
- softmax normalizes all attention weights
- The result is the weighted sum of all value vectors.

The transformer module results in a high-dimensional feature map, which is a global context.

#### 3.3.4 Leaf texture attention module (LeafATM)

**Figure 5:**
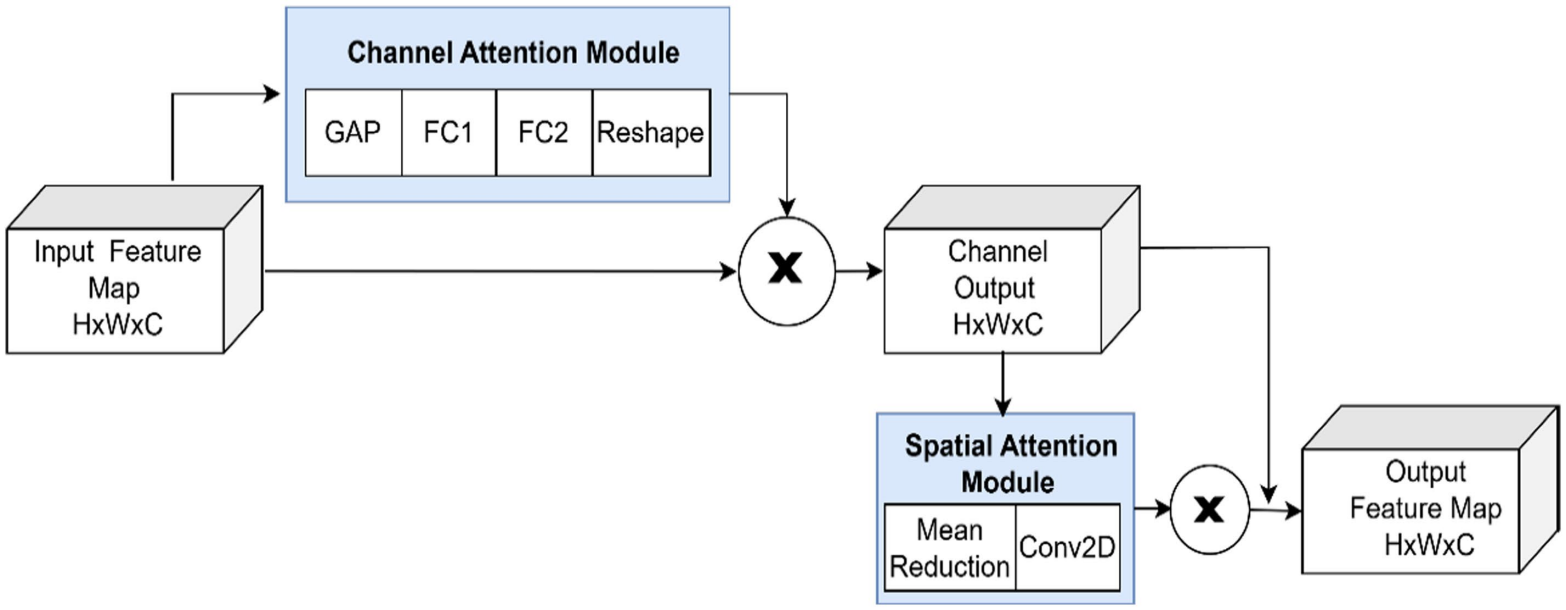
Leaf texture attention module.

The integration of both channel and spatial attention mechanisms is inspired by their complementary roles in feature refinement. Channel attention learns to weigh feature channels that are most informative for disease classification, effectively learning what features to focus on. Spatial attention then determines where in the feature map these important patterns are located. This dual mechanism can be effective in attention-based models for attention-based plant disease detection, where channel-wise attention via squeeze-and-excitation blocks led to better classification accuracy. The design of the sequential use of channel attention and spatial attention is a conscious choice: the channel attention is used to refine the representation of the features first by weighing different feature channels, then the spatial attention works on the refined representation to select the spatially relevant regions. This ordering has been determined to be effective when designing an attention mechanism. The LeafTAM module adds extra features to the CNN feature map by using dual attention mechanisms including channel and spatial attention as shown in Figure 5.

##### 3.3.4.1 Channel attention

Channel attention employs a two-layer Multi-Layer Perceptron (MLP) with a bottleneck structure to learn non-linear relationships between channels. The bottleneck design (controlled by a reduction ratio) reduces the dimension in the intermediate layer, then expands back to the original channel dimension. This compression forces the network to learn compact, essential inter-channel relationships rather than memorizing channel correlations, thereby improving generalization and reducing the risk of over fitting.

Global average pooling is applied across each channel of the feature map to create a channel descriptor. This is passed through two dense layers (with a bottleneck defined by a reduction ratio) and activated using a sigmoid function. The resulting attention weights are applied multiplicatively to the input feature map, emphasizing more informative channels.

Given a feature map F *ϵ* R^HxWxC^, global average pooling(*z_c_*) is applied as shown in Equation 8.


$$z_{c}=\frac{1}{\mathrm{HX}\;W}\;\sum_{i=1}^{H}\;\sum_{j=1}^{W}F_{i,j,c}$$ (8)

Where,

H and W represent the spatial height and width of the feature map.

C denotes the number of channels.

A two-layer MLP follows as given in Equations 9, 10.


$$a_{c}=\sigma (W_{2}.\text{RELU}(W_{1}.z)),\;\;\;\;a\in {\mathbb{R}}^{C}$$ (9)

$$F^{\prime }=F⊙a$$ (10)

where a_c_ denotes the channel attention vector, W_i_ are learnable weight matrices, Z represents reduction ratio, RELU(.) is the Rectified Linear Unit activation function, *σ*(.) denotes the sigmoid activation function, ⊙and denotes elementwise (Hadamard) multiplication.

##### 3.3.4.2 Spatial attention

After channel enhancement, spatial attention is applied by computing the channel mean and convolving it with a 7 × 7 filter. The 7 × 7 kernel provides sufficient receptive field to capture disease symptom regions that may span multiple pixels while remaining computationally feasible. This results in a spatial attention map, that highlights important regions within the feature map and ensures that the model focuses on leaf areas associated with the disease patterns.

After channel enhancement, the spatial attention is computed as shown in Equations 11, 12.


$$M_{s}=\sigma \;(\;\mathrm{Con}v_{7x7}(\mathrm{Mea}n_{\text{channel}}(F^{\prime })))$$ (11)

$$F^{\prime \prime }=F^{\prime }⊙M_{s}$$ (12)

Then, the output $F^{\prime \prime }$ is pooled globally as described in Equation 13.


$$\mathrm{TA}M_{\text{features}}=\mathrm{GAP}(F^{\prime \prime })$$ (13)

The basic output from LeafTAM is then globally pooled and added to the feature ensemble. Each feature stream is processed using the Global Average Pooling before being concatenated. This has several benefits: (1) it reduces dimensions of each feature map to a fixed length vector irrespective of the dimensions of the feature maps, hence it is possible to perform feature fusion among features from different architectural branches, (2) it drastically reduces the dimensionality of the concatenated feature vector, thereby preventing parameter explosion in the classification head, and (3) it provides inherent regularization, which helps improve generalization.

##### 3.3.4.3 Feature fusion and classification

The pooled outputs of the CNN branch, the ViT branch, and the LeafTAM-enhanced CNN feature are then concatenated together to form a unified feature vector containing multi-scale and context-aware information as represented in Equation 14. This design is motivated by the hypothesis that different feature streams capture complementary information:

- CNN features capture general local spatial patterns from raw inputs
- LeafTAM features emphasize texture-oriented disease patterns with selective attention
- ViT features capture global contextual dependencies across the entire leaf surface

By maintaining these as independent streams before fusion, the model can learn specialized representations in each pathway before combining them into a unified feature vector. The dimensionality of all three feature streams is preserved by applying a concatenation function, which allows the subsequent classification layers to learn optimal weightings and combinations of features from each stream. In contrast, element-wise operations such as addition and multiplication require features to have identical dimensions and may lose information through premature combination.


$$Z=\text{Concat}(\mathrm{CN}N_{\text{features}},\mathrm{Vi}T_{\text{features}},\mathrm{TA}M_{\text{features}})$$ (14)

This vector is passed through a two-layer fully connected classification head, which learn nonlinear decision boundaries in fused feature space. It has a 512-unit dense layer with ReLU, which learns high-level abstractions from the concatenated features, batch normalization, dropout, and a 256-unit dense layer with ReLU, which performs dimensionality reduction, batch normalization, and dropout.

Batch normalization has the ability to stabilize training and improve generalization. Dropout layers are incorporated to prevent over fitting. This regularization technique has been exercised in plant disease detection models to improve test data generalization.

Finally, a softmax layer predicts the class label among the 38 plant disease categories. Softmax is the standard choice for multi-class classification tasks to optimize the model’s ability to assign correct class labels.

#### 3.3.5 Loss functions

Sparse categorical cross-entropy loss function is used for efficient multi-class classification with integer labels as expressed in Equation 15.


$$L(y,\hat{y}\;)=-\log({\hat{y}}_{y})$$ (15)

## 4 Experimental setup

### 4.1 Evaluation protocol

This study employs a single train-validation-test split rather than k-fold cross-validation due to the substantial size of the dataset. The dataset was partitioned using stratified sampling to maintain class distribution proportions across all splits: Test Set (20%) is reserved through stratified random sampling, Training Set (64%) is used for model training, and the remaining 16% are used as the Validation Set.

The stratified sampling approach guarantees that all the 38 disease classes are represented in training, validation and test data according to their original distribution in the PlantVillage dataset, preserving the class proportions, even for minority classes (e.g., Potato_healthy with only 30 test samples will maintain representation across all splits). Modern deep learning practice increasingly favors single train-validation-test splits for large-scale datasets, where the size of held-out test data will provide sufficient statistical power to evaluate generalization performance. In contrast, *k*-fold cross-validation tends to be used for smaller data sets in which repeated evaluation of different data subsets is required to ensure generalizability. In addition, the single-split approach is a reasonable trade-off between robustness validation and computational feasibility. The three-way split (train-validation-test) makes sure the test set is not seen at all during training. Stratified sampling across both train-validation and validation-test splits ensures that the class distribution proportions are preserved across all partitions of data. Stratification ensures that the minority classes are properly represented in all splits, avoiding cases where rare classes may be completely missing from the training or test sets because of random sampling. Moreover, the training process has Early Stopping as Generalization Safeguard. Augmentation acts as an implicit regularization technique that helps to improve generalization beyond the training distribution and robustness.

### 4.2 Training configuration

The model was trained using the Adam optimizer with a learning rate (lr) of 1e-4. The loss function used is Sparse Categorical Cross-Entropy. The Adam optimizer was used because of its adaptive per-parameter learning rates (Kingma and Ba, 2014) that effectively manage the visual properties and symptom multiplicity of the 38 different classes of the 38 different plants in the PlantVillage dataset, and its ability to remain stable on the large-scale dataset with more than 54,000 heterogeneous images. The best objective function to use in this multi-class, single-label classification problem was categorical cross-entropy loss, which effectively maximizes the probability of prediction in the correct disease category and reduces the probability of the competing classes, which would naturally fit the mutually exclusive nature of plant disease diagnosis, in which a leaf is either healthy or diseased.

Training was conducted for 50 epochs with a specified batch size of 16 in a Kaggle cloud computing environment with an NVIDIA Tesla P100 GPU with 16 GB VRAM. The model was implemented in Python using Tensorflow deep learning framework. To enhance training efficiency and prevent over fitting, the EarlyStopping technique was used with patience of 5 epochs, and ModelCheckpoint was implemented to save the best model based on validation loss. In addition, a custom EpochCheckpoint callback was used to resume training from the last completed epoch.

### 4.3 Performance metrics

The model’s performance is evaluated using standard metrics, namely precision, recall, F1-score, and accuracy as shown in Equations 16−18.

#### 4.3.1 Precision

Precision measures the proportion of exactly identified diseased leaves to the number of leaves predicted as diseased in total. It indicates how accurately the model classifies a plant as infected.


$$\text{Precision}=\frac{\mathrm{TP}}{\mathrm{TP}+\mathrm{FP}}$$ (16)

#### 4.3.2 Recall

(Sensitivity or True Positive Rate) measures the quantitative share of exactly identified diseased leaves and total actual diseased leaves. It shows the model’s ability to identify infected plants without missing any cases.


$$\text{Recall}=\frac{\mathrm{TP}}{\mathrm{TP}+\mathrm{FN}}$$ (17)

#### 4.3.3 F1-score

Is the harmonic mean of precision and recall. It offers a balanced measure of a model’s ability to classify diseased leaves accurately with reduced false positives and false negatives.


$$F1\;\text{score}=2\;X\;\frac{\text{Precision}\;X\;\text{Recall}}{\text{Precision}+\text{Recall}}$$ (18)

Where,

TP acts as True Positives, FP acts as False Positives, FN acts as False Negatives, and TN acts as True Negatives.

#### 4.3.4 Receiver operating characteristic (ROC) curve and area under curve(AUC) analysis

ROC and AUC metrics are employed to assess the efficacy of the proposed model in identifying various plant diseases. The ROC curve is used to depict the capability of the model to differentiate diseased leaf subsets by plotting the True Positive Rate (TPR) versus False Positive Rate (FPR) over a range of thresholds. Although ROC analysis is typically applied to binary classification to classify diseased and healthy plants, it can be made to operate in a multi-class framework, which is to identify multiple plant diseases. In this strategy, an independent ROC curve is developed against each type of disease and against all the other diseases and healthy classes.

- An AUC of 1.0 denotes perfect separation, i.e., the model correctly distinguishes a specific plant disease from all others without error.
- An AUC of 0.5 suggests the model is making random guesses and cannot differentiate between leaf conditions.

In multi-class plant disease detection, two common ways to compute overall AUC are:

- *Micro-Averaged AUC*: It aggregates all TP and FP across all leaf classes. This method is influenced by class size, so disease types with more leaf images have more weight.
- *Macro-Averaged AUC*: It calculates AUC for each disease class separately and then averages them, treating all disease types equally. This approach is more appropriate when the dataset is imbalanced across crop types or disease classes.

## 5 Experimental results and discussion

To assess the performance, the models were trained and tested on the PlantVillage dataset, using a train-validation-test split of 64:16:20. The performance indicators for all models were computed on the test set consisting of 10,861 images across 38 classes.

### 5.1 Quantitative evaluation

**Figure 6:**
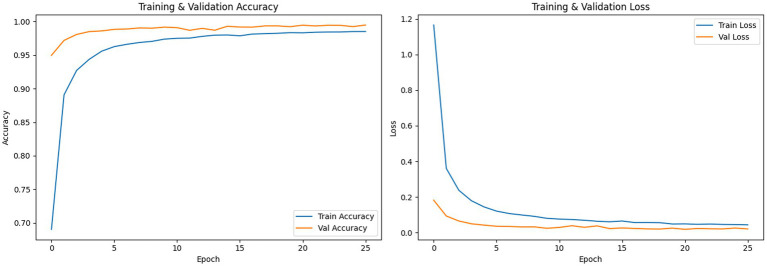
Training and validation accuracy and loss curves for LeafFusionNet.

The suggested model consists of CNN, ViT, and LeafATM modules. In addition, it improves the architecture with the introduction of a layer of texture preprocessing by Gabor filters. The model was capable of obtaining a training loss of 4.89 and an accuracy of 98.33 in the training. The highest validation accuracy was 99.42 and the validation loss of the same was 2.01. Its test accuracy was 99.33% and test loss was 2.39 on the test set. The macro average F1-score and the weighted average F1-score were 0.99 each. Figure 6 includes the training and validation accuracy/loss curves. Such graphs are useful in visualizing the learning advancement of the LeafFusionNet model as well as deciding on whether the model is over fitting or under fitting. The accuracy curve in training demonstrates the rapid improvement at the beginning, with the accuracy starting with approximately 70 percent in epoch 1 to the highest accuracy, where it reaches 95 percent in epoch 5, which implies that the model can learn the discriminative features in the plant disease classification task in a rather short period. This fast initial learning behavior is characteristic of transfer learning situations where the pre-trained ViT component is able to learn good initial feature representations by which it can converge more quickly than a random initialization. In the subsequent epochs, training accuracy was gradually increasing and 98.33% with the stop of training.

Correspondingly, the training loss curve shows the phenomenon and declines steeply at the beginning (at approximately 1.2 at epoch 1 to 1.2 at epoch 2) and then becomes gradually more and more lower to reach its endpoint of 4.89% (0.0489). The monotonic drop in training loss with no irregular changes also indicates that the gradient-based optimization with the Adam optimizer is stable, which indicates that the learning rate (1e-4) and the batch size (16) are being used in a well-adjusted manner in this task. Significantly, the validation accuracy curve follows the training accuracy closely during the process, and it will soar at the initial stages of the process, and ultimately, it will reach 99.42 percent at the optimum checkpoint. The validation curve is consistently above or very close to the training curve for most of the training processes, which is a very unusual but positive pattern. This behavior suggests that the model generalizes very well to unseen validation data—in fact, a little better than the model’s performance on training data. The validation loss curve also shows good generalization, which drops from about 0.15 at epoch 1 to 2.01% (0.0201) at the best checkpoint. The fact that training loss (4.89%) is close to validation loss (2.01)—validation loss is actually lower than training loss—is a good indication that we are not over fitting.

The training dynamics that are observed clearly show that the model is appropriately fit without significant overfitting or under fitting. The accuracy of the training (98.33%) and validation (99.42%) is very close to each other, a difference of 1.09 percentage points, and the validation accuracy is greater than the training accuracy. This almost zero or negative generalization gap is the converse of the overfitting trend, where the training performance is significantly higher than the validation performance. The training loss (4.89) is higher than the validation loss (2.01), indicating that the model is not learning training-specific patterns at the expense of generalizing. The validation accuracy curve does not assume the characteristic rise, then fall trend of overfitting, where validation accuracy is increasing but becomes worse as the model becomes increasingly over fitted to the training data. Rather, the accuracy of validation rises by a constant rate, and it is at a high level with no further degradation.

**Figure 7:**
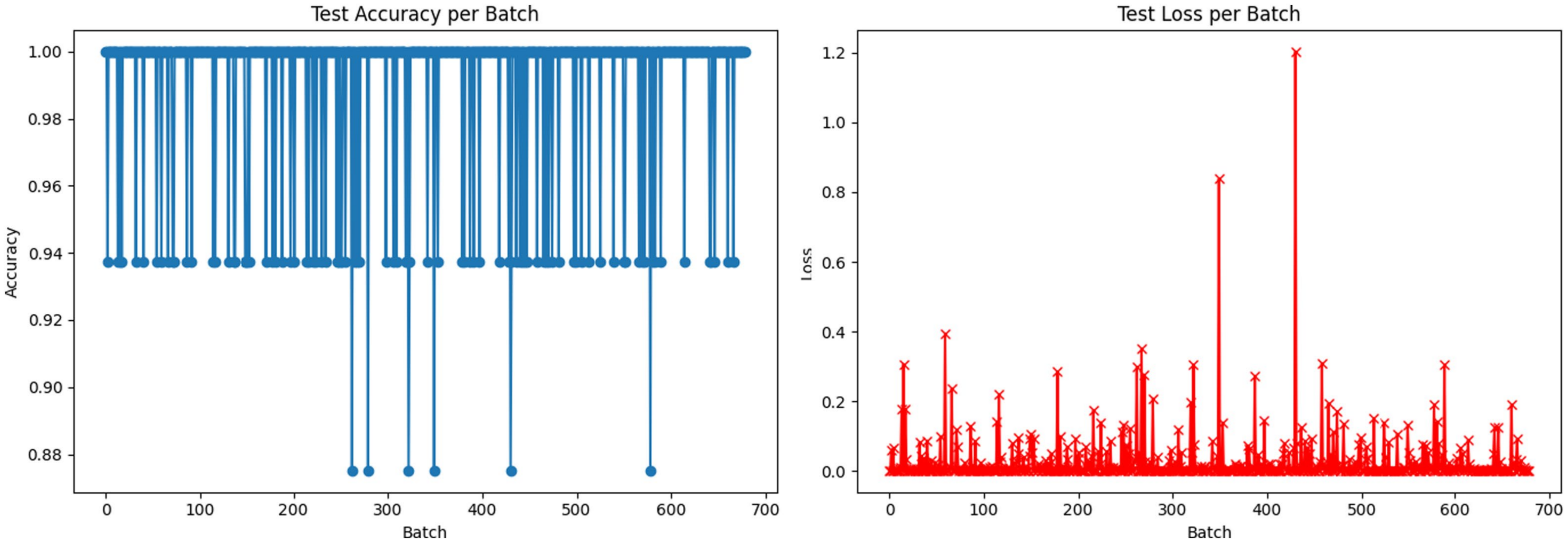
Test accuracy and loss per batch for the LeafFusionNet.

The plotted graphs show that there is a rise in the training and validation accuracy without any significant deviation, which may signify stable learning. Besides, test accuracy and test loss graphs, as in Figure 7, were created batch-wise to check the consistency of the model across batches and visualize whether there were any drops in performance, which would be isolated or consistent.

As a result, it can be observed that the test accuracy was consistent across batches with only minor fluctuations, which supports consistent model behavior. The value of losses could be high occasionally in some test batches, probably because of challenging samples or bias in classes.

**Figure 8:**
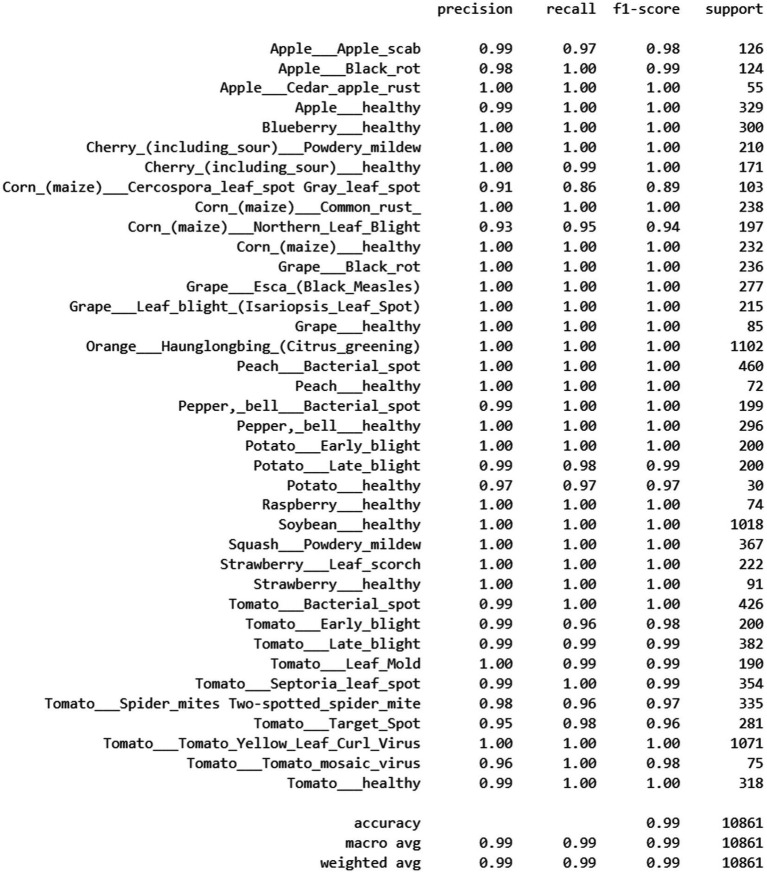
Classification report for the proposed model LeafFusionNet.

The model had high performance in terms of precision, recall and F1 Scores (1.00) in most classes, including such major crops as Tomato Yellow Leaf Curl Virus, Soybean healthy and Citrus Greening. Conversely, the performance of certain crops such as Corn_(maize)_Cercospora_leaf_spot (F1-score: 0.89) and Corn_(maize)_Northern_Leaf_ Blight (F1-score: 0.94) declined by a small margin. These findings in general indicate very good generalization and strength across all categories of 38 plant diseases in the data set. All the classification report on the proposed model is illustrated in Figure 8. Many classes that are underrepresented have, as shown in Figure 8, a perfect or near-perfect F1-score despite gross imbalance. For example:

- Potato_healthy (30 samples, smallest class): Precision = 0.97, Recall = 0.97, F1 = 0.97
- Apple_Cedar_apple_rust (55 samples): Precision = 1.00, Recall = 1.00, F1 = 1.00
- Raspberry_healthy (74 samples): Precision = 1.00, Recall = 1.00, F1 = 1.00

This demonstrates that the model successfully learns discriminative features for minority classes without requiring explicit balancing interventions.

**Figure 9:**
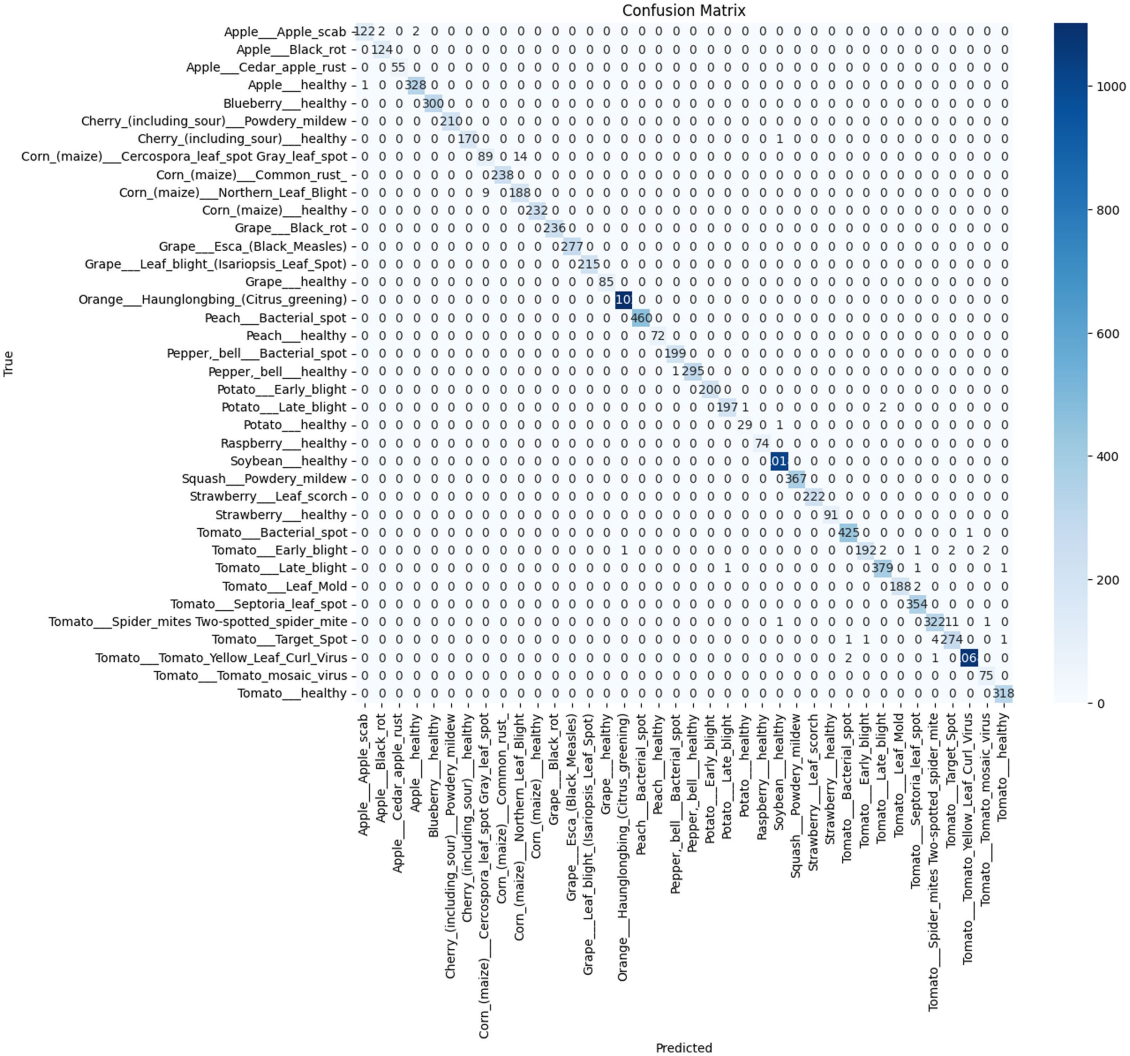
Confusion matrix for the proposed model LeafFusionNet.

The confusion matrix in Figure 9 highlights this improvement, particularly in texture-heavy classes. This matrix demonstrates strong performance, but with minor differences in misclassification patterns.

It is observed that the medium-sized classes have a low F1 score, not among the smallest which indicates the imbalance nature of dataset does not affect the prediction.

The two classes with lowest F1-scores are:

- Corn Cercospora leaf spot (103 samples, F1 = 0.89)
- Corn Northern Leaf Blight (197 samples, F1 = 0.94)

This suggests that performance degradation is driven by visual similarity between corn diseases rather than insufficient training data. The confusion matrix confirms minor confusion between these corn disease classes, likely due to overlapping symptom patterns (both present as leaf spotting/blighting with similar texture characteristics).

**Figure 10:**
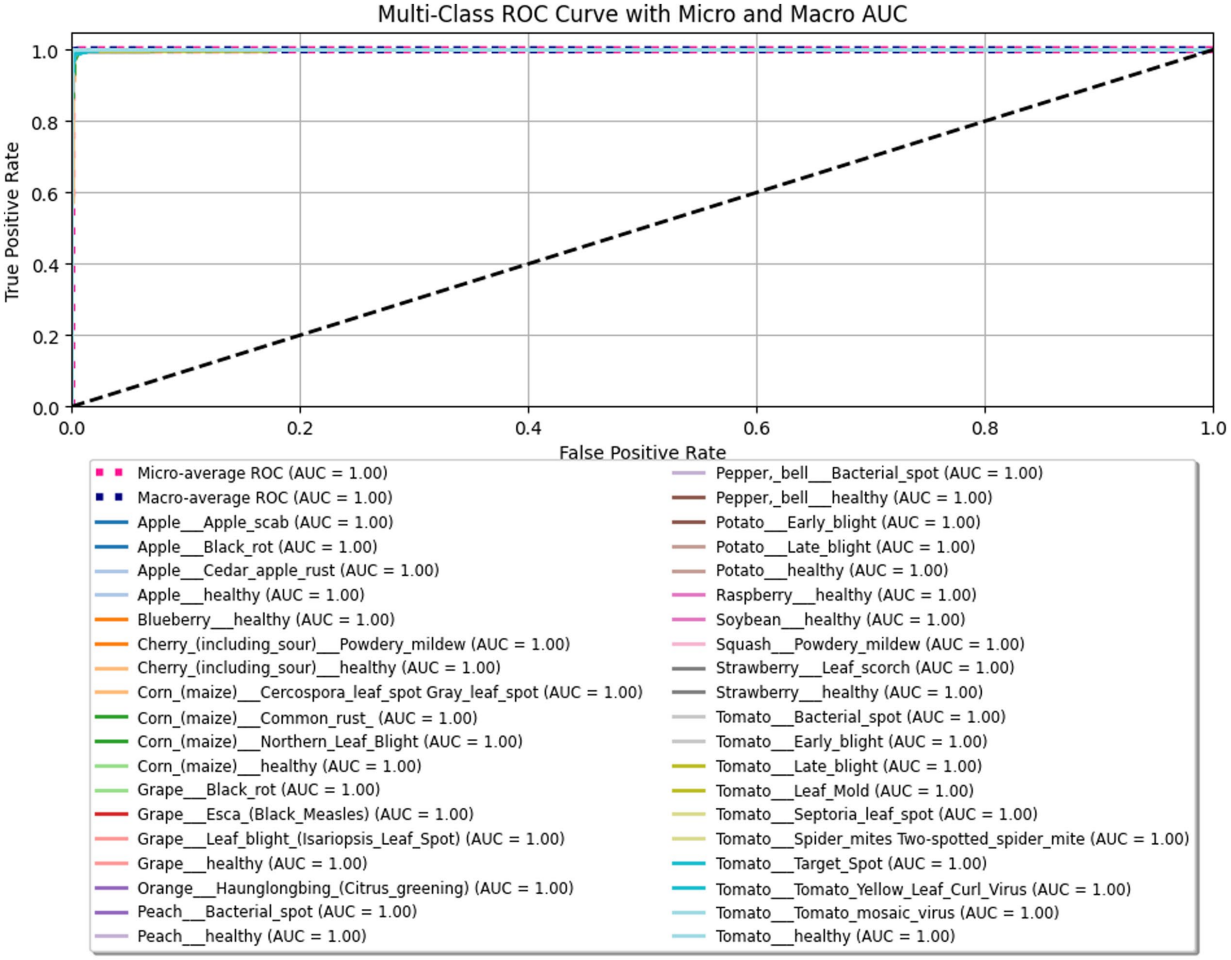
Multi-class ROC curve for the proposed model LeafFusionNet, showing the individual class-wise curves along with the micro and macro-averaged ROC curves.

The ROC curves for each class are shown in Figure 10, along with aggregated micro and macro curves. All curves lie close to the top-left corner of the graph. This further validates the model’s robustness and reliability.

### 5.2 Comparative analysis

#### 5.2.1 Ablation study results

The ablation experiment determines the cumulative role of the incorporation of various architectural elements to the plant disease classification system as tabulated in Table 2. The original basic CNN model attains an accuracy of 87.97%, which means that traditional convolutional features are not enough to represent the complex changes in vegetative disease patterns. The introduction of the Vision Transformer (ViT-base) brings the accuracy to 93.9 percent, showing that transformer-based global feature extraction has a high potential in modeling the long-range dependencies.

**Table 2:** Ablation study results of different components.

Modelsa	Accuracy (%)	Precision	Recall	F1 score
Basic CNN	87.97	0.88	0.81	0.83
ViT(base)	93.9	0.91	0.96	0.93
ViT + CNN + LeafTAM(without gabor)	98.97	0.99	0.99	0.99
Proposed model/LeafFusionNet	99.33	0.99	0.99	0.99

Additional ViT and CNN with LeafTAM attention module (excluding Gabor features) results in a significant improvement in performance with 98.97% accuracy. This demonstrates that LeafTAM is effective in terms of refining features by giving more priority to disease-relevant regions. Lastly, the accuracy of the proposed model, based on the use of Gabor filters in combination with ViT, CNN, and LeafTAM, is the highest, 99.33%. Gabor filters add extra texture-level discrimination to enable the model to better learn the pattern of veins of leaves, lesion textures, and orientation-specific disease information. The ablation results prove that each additional component will improve the performance, and the entire integrated architecture will provide the strongest and most discriminative representation of the feature to classify the plant diseases.

Both models demonstrated excellent generalization and classification capabilities, with minimal overfitting. This model outperformed slightly in overall accuracy and test loss and provided better robustness to texture-heavy disease classes, aligning with the hypothesis that Gabor filtering enhances the capture of structural detail. Thus, the model may offer a more balanced trade-off in scenarios with varied leaf textures.

As a single real-time agricultural advisory system, it was preferred to use a unified model, rather than plant-wise independent models, since the system is in need of a single scalable solution which can operate with various crops. Deep convolutional networks prove useful when it comes to hierarchical features automatically extracted by the networks that identify the plant identity and disease-characteristic features.

There is an average level of class imbalance, with some disease categories having more samples than others. To avoid bias against the majority classes, the use of data augmentation and class-balanced sampling were implemented during training. Moreover, the evaluation of performance was considered with precision, recall, and F1-score, as well as the overall accuracy, which is more accurate with unequal conditions.

Although the overall accuracy was high, it is worth noting that the accuracy per se might not be a full indication of the performance in the minority classes. The confusion matrix reveals that few diseases that are under-represented have a relatively lower recall, which opens the opportunities of expanding the dataset in the future.

#### 5.2.2 Comparison with state-of-the-art models

To validate the efficacy of the proposed model, its performance was compared with several state-of-the-art disease detection methods using the Plant Village dataset, as tabulated in Table 3. Jiang et al. (2025) achieved an accuracy of 93.53 percent, indicating reasonable classification capability but limited robustness. The models proposed by Alhwaiti et al. (2025) with the YOLO architecture showed better results. YOLOv3 reached 97% accuracy, and YOLOv4 attained an accuracy of 98%, supported by high precision and recall values. Similarly, Isinkaye et al. (2025) also presented impressive results with an accuracy of 96.8%, while Hassan et al. (2021) obtained an accuracy of 97.02%, but without detailed metrics for precision and recall.

**Table 3:** Comparison of LeafFusionNet with other models.

Author/reference	Model	Train test split	Accuracy (%)	Precision (%)	Recall (%)	F1 score (%)
Jiang et al. (2025)	PlantCaFo	Custom	93.53	—	—	—
Alhwaiti et al. (2025)	YOLOV3	80/20	97	97	98	96
	YOLOV4	80/20	98	98	99	99
Isinkaye et al. (2025)	Hybrid	80/20	96.8	94.2	98.1	96.1
Hassan et al. (2021)	MobileNetV2	80/20	97.02	—	—	—
Albahli (2025)	AgriFusionNet	80/20	94.3	94.1	93.9	94
Our proposed model	LeafFusionNet	80/20	99.33	99	99	99

In contrast, the proposed LeafFusionNet model outperforms all the existing approaches with a higher accuracy of 99.33% along with 99% precision, recall, and F1-score. This significant improvement proves that the integration of transformer-based global attention, CNN-driven local feature extraction, texture-aware Gabor filtering, and the LeafTAM attention module is very effective.

The train test split used by most of the state-of-the-art model is 80:20. Whereas studies based on benchmarking, like those by Alhwaiti et al. (2025) and Isinkaye et al. (2025), use specialized preprocessing pipelines, such as Gaussian denoising, Canny edge detection, and handcrafted feature fusion (HOG/GLCM), our proposed framework employs a simplified end-to-end pipeline, which is comprised of standard normalization and resizing, and has a high architectural capacity to extract discriminative features directly in raw pixel data. The differences in preprocessing techniques between this study and the mentioned baselines provide additional evidence of the proposed model’s strength. The model’s ability to outperform frameworks that use advanced image enhancement suggests a superior capacity for learning spatial hierarchies, effectively bypassing the need for manual feature engineering.

### 5.3 Grad-CAM visualization and interpretability

**Figure 11:**
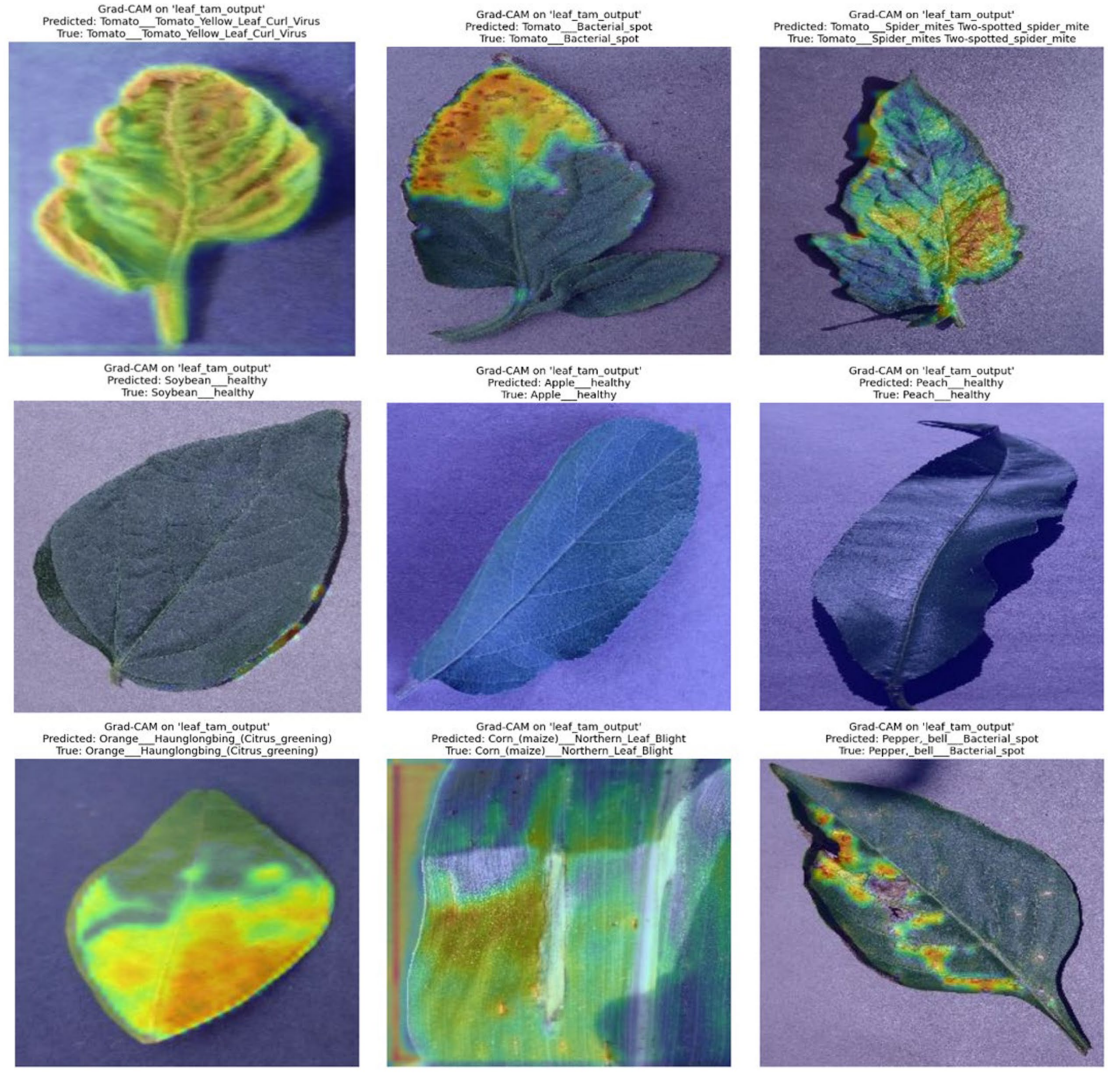
GradCAM visualization results of the proposed model LeafFusionNet.

To evaluate the interpretability of both models, Gradient-weighted Class Activation Mapping (Grad-CAM) technique was used. This technique visualizes class-discriminative regions in the input image, helping to understand which parts of the leaf image the model focuses on during prediction. It is useful in verifying whether the model’s attention aligns with actual disease-affected regions. Red, yellow, and orange colors specify that the model focuses strongly on those portions. Blue and red color specifies that less attention is provided by the model in those areas. The GradCAM visualization results of the proposed model are depicted in Figure 11.

The Grad-CAM application on the proposed model produced visual outputs consisting of the ground truth class label, the model’s predicted class, and an overlaid Grad-CAM heatmap on the input image, and a concise explanation of the model’s attention. These visualizations were generated using the LeafTAM layer, which integrates spatial and texture-based attention after the Gabor filter and CNN-ViT fusion.

In the correctly classified ones, the Grad-CAM heatmaps identified biologically relevant parts of the leaf images. High attention, in the form of red and yellow, was frequently centered on diseased regions (in the form of visible lesions, discolored spots or structurally abnormal veins). To obtain healthy leaves, despite a general trend of more diffuse patterns indicated in heatmaps, attention was usually concentrated in the areas of the structure that are relevant in terms of assessment of the base, namely, leaf edges or midribs. In cases where the prediction was incorrect or showed borderline confidence, the heatmaps often revealed misaligned or fragmented focus. Instead of tightly concentrating on specific symptomatic regions, the attention was more scattered or shifted to irrelevant portions of the leaf. These observations, directly visible in the Grad-CAM heatmap produced, suggest that although the LeafTAM module is effective in many cases, its interpretation of fused Gabor-texture and spatial cues may require further refinement.

## 6 Conclusion

The LeafFusionNet model demonstrated high performance on the PlantVillage dataset with high accuracy and interpretable visual explanations using Grad-CAM. The attention maps demonstrated that both models are generally able to give a strong performance in focusing on remarkable regions of infected or healthy leaves, hence assessing the efficiency of the architecture and attention mechanisms involved. The texture enhancement using a Gabor filter layer gave slightly more localized attention in some of the disease categories (especially where fine-grained patterns played a major role). However, limitations in dealing with certain ambiguous or borderline cases demonstrated the possibility for further improvement in both models.

Future research could be extended with the proposed model by applying real datasets with field images of plants captured in different environmental conditions. This would also verify the generalization ability outside the controlled environment of the PlantVillage dataset. Moreover, some other methods of explain ability, like LIME (Local Interpretable Model-Agnostic Explanations) (Ribeiro et al., 2016) or SHAP (Shapley Additive Explanations) (Lundberg and Lee, 2017), may give a more informative view on the importance of features and decision boundaries, complementing Grad-CAM images. Further model robustness against typical field effects, such as lighting, viewing angle, clutter in the background, and occluding objects, may also provide better practical implementation in the agricultural environment.

## Data Availability

Publicly available datasets were analysed in this study. This data can be found here: https://www.kaggle.com/datasets/mohitsingh1804/plantvillage.
